# Laminin-511 and integrin beta-1 in hair follicle development and basal cell carcinoma formation

**DOI:** 10.1186/1471-213X-10-112

**Published:** 2010-11-10

**Authors:** Mindy C DeRouen, Hanson Zhen, Si Hui Tan, Samantha Williams, M Peter Marinkovich, Anthony E Oro

**Affiliations:** 1 Cancer Biology Graduate Program 251 Campus Drive, MSOB X234, Stanford, 94305-5173), USA; 2 Program in Epithelial Biology, Stanford University, School of Medicine, CCSR 2145c, 269 Campus Drive, Stanford, CA 94305, USA; 3 Dermatology Service, Palo Alto VA Medical Center, 3801 Miranda Ave Palo Alto, California 94304, USA

## Abstract

**Background:**

Initiation of the hair follicle placode and its subsequent growth, maturation and cycling in post-natal skin requires signaling interactions between epithelial cells and adjacent dermal cells and involves Shh signaling via the primary cilium. Previous reports have implicated laminins in hair follicle epithelial invagination.

**Results:**

Here we use a human BCC model system and mouse mutants to re-evaluate the role of laminin-511 in epithelial invagination in the skin. Blocking laminin 511 and 332 in BCCs maintains primary cilia and Shh signalling, but prevents invagination. Similarly, in laminin-511 and dermal beta-1 integrin mutants, dermal papilla development and primary cilia formation are normal. Dermal beta-1 integrin mutants have normal hair follicle development.

**Conclusions:**

Our data provides support for a primary role of laminin-511 promoting hair follicle epithelial downgrowth without affecting dermal primary cilia and Shh target gene induction.

## Background

Hair follicle morphogenesis requires signaling interactions between epithelial cells and adjacent dermal cells that form the specialized mesenchyme called the dermal papilla. During these processes, Sonic hedgehog (Shh) signaling is required in both the epithelial and dermal compartments. Inappropriate epithelial Shh target gene induction is sufficient to cause basal cell carcinoma (BCC), one of the most common tumors in Caucasians, with an incidence of over a million cases per year in the U.S. BCCs also have a striking reliance on adjacent stroma for continued growth and invasion, implying that non-cell autonomous factors influence the extent of Shh target gene induction in tumors [1-3]. Despite extensive study, factors that regulate epithelial-mesenchymal interactions during normal or neoplastic epithelial growth remain poorly understood.

The primary cilium is a microtubule-based organelle crucial for the regulation of Shh signalling and growth (for reviews see [4-7]). Receptors positioned within the cilium transduce signals through transcription factors that are activated directly in the cilium or in the cell body. Mutations giving rise to defective primary cilia or improper placement of signaling molecules within the cilium result in a plethora of clinical manifestations [8,9]. In particular, mutations in genes encoding intraflagellar transport proteins impair Shh signaling and result in limb bud and neural tube defects similar to those seen with inactivating mutations of the Shh pathway [10-14]. Two recent reports describe requirements for IFT88, an intraflagellar transport component, and Missing in Metastasis, an actin regulatory protein, for Shh signalling in the dermal papilla and depict a necessary role for the primary cilia in hair follicle development [15,16]. Moreover, a requirement for the primary cilia in BCC formation has been demonstrated, which reinforces the importance of the primary cilia in Shh signaling and epithelial downgrowth [17].

Laminin-511, a basement membrane zone protein, is also required for hair follicle downgrowth. Laminin-511 (LM511) and laminin-332 (LM332) are the most abundant laminins in developing and adult skin. Both are secreted from keratinocytes and incorporate into the basement membrane zone of hair follicles and interfollicular epithelia, but LM511 is particularly abundant in the basement membrane zone surrounding hair follicles (for a review see [18]). Laminins also signal intracellularly through transmembrane receptors, including the integrin family of heterodimers. In the skin epithelium, the most abundant LM511 receptor pair is integrin alpha-3, beta-1. LM332, on the other hand, predominantly associates with integrin alpha-6, beta-4 in hemidesmosomes [19]. These interactions have only been described, however, for integrin receptors within epithelial cells, not dermal papilla cells.

Mice null for the alpha 5 subunit of LM511 (lamα5-/- mice) show arrest of hair follicles during the hair germ stage of hair follicle morphogenesis. Arrested follicles resemble *SHH*-null follicles, indicate reduced expression of *SHH *and *GLI1 *in hair follicle epithelia and have a reduction in epithelial proliferation [20]. Furthermore, previous results indicated that primary cilia are absent in the dermal papilla of lamα5-/- mice at E16.5 [21]. These and additional observations suggested that epithelial derived LM511, through binding integrin beta-1 (Itβ1), maintains the primary cilia in dermal papilla cells and is therefore required for Shh ligand reception in those cells.

In contrast, epithelial-derived laminins are also known to act directly on the epithelium to promote invasion. Developmental studies with conditional mutants of epithelial Itβ1 demonstrate its role in epidermal/dermal attachment and proper hair follicle development [22,23]. In human graft models of SCC, epithelial downgrowth depends on both Itβ1 and LM332, which indicates a need for both adheshion and signaling for continued epithelial growth [24].

Here we use a human BCC model system and a conditional mouse mutant lacking Itβ1 in dermal mesenchyme to re-evaluate the role of LM511 in epithelial downgrowth. We provide evidence that LM511 interaction with integrin β1 in epithelium, rather than with dermal papilla, is required for hair follicle invagination.

## Results

### LM511 and LM332 blockade inhibits BCC invasion but not Shh signaling

Human skin xenografts overexpressing Shh ligand provide an excellent model to study in vivo BCC lesion formation [25]. We used antibody blocking reagents in conjunction with this model to determine whether laminin function is required for BCC formation in these grafts. The 4c7 antibody binds the LM511 globular domain and thereby inhibits integrin binding [26]. The BM165 antibody was raised against the α3 subunit of LM332[27]. For experiments receiving a single antibody (IgG, 4c7 or BM165) animals received intraperitoneal injections carrying 1.0 mg of antibody starting in the first week of growth and then every week thereafter. In a preliminary experiment, we determined that blocking grafts with both LM511 and LM332 antibodies in the first week of growth results in severe blistering, so in this case we began injections in the second week of growth. Some grafts, especially those treated with BM165 to block LM332 function (starting in the first week of grafting), were lost due to the formation of scar tissue in the graft site [28].

**Figure 1:**
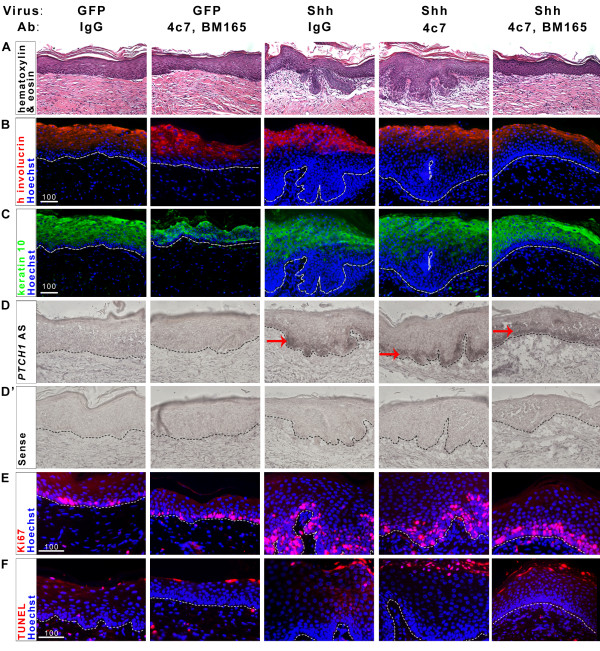
**LM511 and LM332 inhibition reduces tumor invasion in human BCC skin xenografts**. A: Hematoxylin and eosin-stained sections of human skin xenografts carrying either control GFP or *SHH *retrovirus and treated with control IgG, 4c7 antibody against LM511 or BM165 antibody against LM332, as indicated. Images captured at 20x magnification. B: Indirect immunofluoresence for human-specific involucrin (red) confirms the human origin of graft tissue. C: Indirect immunofluoresence for keratin 10 (green) indicates all non-basal cells in graft epidermis. D: DIG RNA in-situ with anti-sense probe to *PTCH1 *transcript in indicated xenografts. Anti-sense probe signal is seen in all *SHH*-expressing grafts. Black dotted lines indicate epidermal/dermal boundary. Red arrows indicate DIG signal. D': DIG RNA in-situ with sense probe to *PTCH1 *transcript in indicated xenografts. Images are 20x magnification. E: Indirect immunofluoresence for Ki67 (red) in indicated graft tissues marks proliferating cells. F: TUNEL-TMR Red staining indicates apoptotic cells in graft tissues. E-F: White dashed lines indicate the epidermal/dermal boundary. Scale bars indicate 100 μm.

*SHH*/IgG grafts form BCC-like lesions as expected (n = 3) and contain thickened epidermis and invaginations with hallmark BCC features including pallisading basal cells and the strong presence of dermal cells at the epidermal/dermal boundary (Figure 1A). This contrasts sharply with the thin, orderly skin present in GFP/IgG control grafts (n = 4). We confirmed that all grafts analyzed contain human skin, and not murine host skin, using species-specific human involucrin antibodies (Figure 1B). We also determined that graft tissues incorporate both human LM511 and human LM332 into the basement membrane zone, exclude mouse LM511 and are targeted by injected blocking antibodies (data not shown). Severe invaginations similar in appearance to those in *SHH*/IgG grafts are also evident in all *SHH*/4c7 grafts (n = 4), indicating that LM511 blockade does not reduce Shh-mediated BCC formation (Figure 1A). On the other hand, blocking both LM511 and LM332 function (n = 4) inhibits the BCC epithelial invagination seen in *Shh*/IgG grafts. In each of the double-blocked grafts, stratified epithelium was present with little if any epithelium progressing past the dermal/epidermal boundary.

Lack of BCC epithelial invasion could be caused by lack of Shh responsiveness or Shh-dependent invasiveness, so we next assessed the Shh target gene induction in double-blocked grafts. As expected, GFP expressing tissues display only a single layer of keratin 10-negative (basal) cells (Figure 1C) whereas those of *SHH*/IgG and *SHH*/4c7 grafts demonstrate extensive basal layer proliferation and invagination. Notably, the epithelium of *SHH*/4c7, BM165 grafts exhibit 2-3 layers of keratin-10 negative cells similar to the keratin-10 expression profile of non-invasive regions of *SHH*/IgG and *SHH*/4c7 grafts. These grafts show additional basal layer proliferation and indicate persistent Shh response in LM511/LM332 blocked tissues.

To probe this issue further, we determined the expression of Shh target genes as well as indicators of epithelial cell proliferation and apoptosis. To determine the extent of Shh target gene induction in the grafts, we performed RNA in-situ hybridization with DIG-labeled RNA probes for *PTCH1*, a direct target of Shh pathway activation. Anti-sense probes for *PTCH1 *show the presence of Shh target gene expression, concentrated in basal cells at the leading edge of epithelial invaginations, in *SHH*/IgG and *SHH*/4c7 grafts (Figure 1D, arrows). Despite the reduced appearance of invaginations in *SHH*/4c7, BM165 grafts, these grafts contain *PTCH1 *transcript concentrated in the basal half of the epidermis. The expression of these genes is specific, as *GFP*-expressing xenografts do not show detectable levels of these transcripts. In addition, control sense probes corresponding to the *PTCH1 *transcript do not provide signal in all grafts (Figure 1D'). We conclude, then, that the lack of epithelial invagination in *SHH*/4c7, BM165 grafts is not due to an inability of the Shh pathway to activate target genes.

Shh signaling has a variety of effects on responding cells in a tumorigenic context, but its most notable effects are an increase in proliferation and inhibition of apoptosis in responsive cells. If laminin blockade reduces Shh target gene induction, it may affect the proliferation or apoptotic index of responsive tissues. Therefore, we determined the extent of proliferation and apoptosis in xenograft lesions. We assessed the extent of proliferation in xenografts with immunofluoresence for Ki67, a marker of cellular proliferation. GFP/IgG grafts contain a limited number of proliferating basal cells, restricted to a single cell layer, in the epidermis (Figure 1E). GFP/4c7, BM165 grafts likewise contain few proliferating basal cells. As expected. *SHH*/IgG and *SHH*/4c7 grafts contain an abundance of proliferating cells within tumor invaginations. Concordant with the histological phenotype, *SHH*/4c7, BM165 grafts contain fewer proliferating cells than *SHH*/IgG or *SHH*/4c7 grafts. However, these tissues contain more proliferating cells than the control GFP/4c7, BM165 grafts and even contain proliferating cells in suprabasal layers of the epidermis, which mirrors the proliferation profile of non-invasive regions of *SHH*/IgG and *SHH*/4c7 tissues (Figure 1E and Additional File 1, Figure S1). This indicates that blockade of LM511 and LM332 in *SHH*-expressing grafts does not affect the increased proliferation of cells experiencing excess SHH ligand.

**Figure 2:**
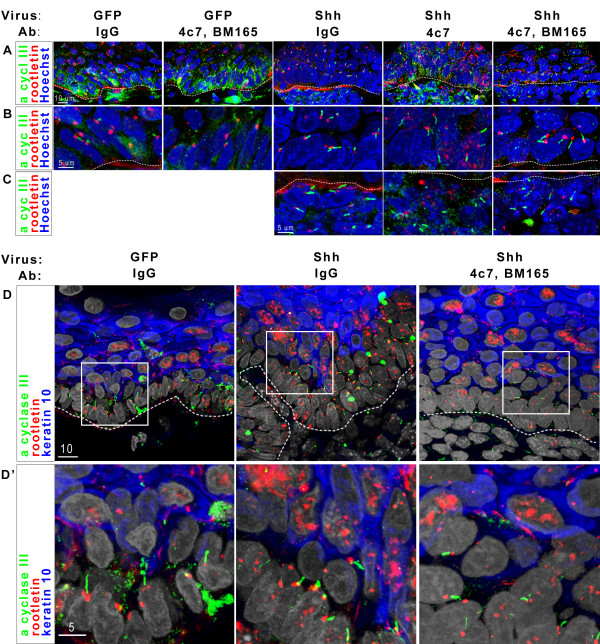
**Xenograft tissues contain primary cilia**. A-C: Indirect immunofluoresence for cilia components. Adenylyl cyclase III (green) indicates cilia shafts, rootletin (red) indicates the basal body at the base of the cilia and Hoechst dye (blue) indicates cell nuclei. A: Cilia are present in both epithelial and dermal cells. B: Zoom of epithelial cells in A. C: Zoom of dermal cells in A. D: Indirect immunofluoresence for cilia components and suprabasal cells. A cyclase III (green) and rootletin (red) mark cilia. Keratin10 (blue) marks suprabasal cells. Hoechst dye (white) indicates cell nuclei. Robust cilia are present only in cells excluding keratin 10. D': Zoom of boxed areas in D. All dashed lines indicate epidermal/dermal boundaries.

In order to determine the apoptotic index in graft tissues, we utilized a fluorescent TUNEL assay. If laminin blockade affects Shh survival signals, we would expect to see an increase in the number of apoptotic nuclei in antibody-treated grafts. This is not the case, as all grafts only show apoptotic nuclei within the stratified layers of the epidermis, not in the relevant basal layer (Figure 2F). We conclude, then, that blockade of LM511 and LM332 in human skin xenografts also does not abolish any anti-apoptotic effects mediated by Shh signalling in epithelial cells.

Our results regarding Shh target gene expression, proliferation and apoptosis in xenograft tissues indicate that antibody blockade of LM511 and LM332 does not drastically reduce Shh response of relevant cells in a tumorigenic context. Since LM511 has been suggested to affect Shh target gene induction in the developing hair follicle via maintenance of the structure of the primary cilium, we also investigated the presence of this structure in our graft experiments.

### Human skin xenografts contain epithelial and dermal primary cilia

Using antibodies to adenyl cyclase III for cilia shaft and rootletin for the basal body, we were able to visualize primary cilia in epithelial cells of all human skin xenografts (Figure 2A and 2B). Furthermore, in *SHH*-expressing grafts, we can also visualize primary cilia in dermal cells adjacent to the basement membrane zone (Figure 2C). Combinatorial staining for keratin 10, which localizes to all suprabasal cells, indicates that the presence of the primary cilia in a cell negatively correlates with keratin 10 expression. Invasions within *SHH*/IgG or *SHH*/4c7 grafts have a greater ratio of keratin 10-negative (basal) epithelial cells to all epithelial cells (Figure 1C), and likewise indicate a large number of cells with primary cilia. Grafts expressing control virus contain only a single layer of keratin 10-negative/cilia-positive cells and exhibit primary cilia only in these cells. Accordingly, non-invasive regions of *SHH*/IgG and *SHH*/4c7 and all regions of *SHH*/4c7, BM165 grafts have 2-3 layers of primary cilia-containing basal cells (Figure 2D and data not shown). So, we can conclude that blocking LM511 alone or blocking both LM511 and LM332 does not ablate primary cilia in epithelial cells or dermal cells adjacent to the basement membrane zone in our xenograft model. We, therefore, also conclude that any negative effect that LM511 and LM332 inhibition has on tumor invasiveness is primary cilia-independent.

### Dermal papilla in hair follicles of lamα5-/- mice appear normal at E16.5

**Figure 3:**
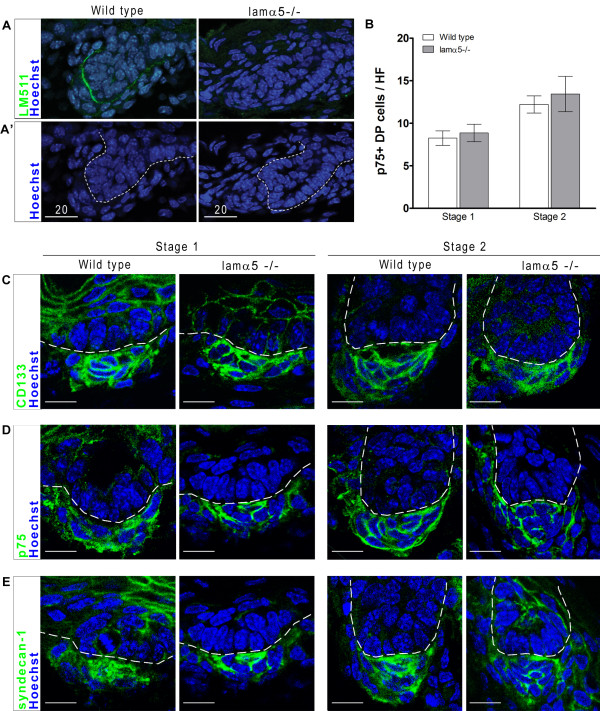
**Laminin alpha 5 null hair follicles normally express dermal papilla markers**. A: Images of indirect immunofluoresence for mouse LM511 (green) in E16.5 wild type and lamα5-/- mice (as indicated) showing a lack of antigen in null animals A': The same images as (A) indicating the Stage 2 hair follicles present in each view. B: Quantitation of p75-positive cells in dermal condensate of Stage 1 and Stage 2 hair follicles of E16.5 wild type and lama5-/- mice. C-E: Confocal images of indirect immunofluoresence for dermal markers CD133 (C), p75 (D) and syndecan-1 (E) in wild type and lamα5-/- Stage 1 and Stage 2 follicles within E16.5 skin. Hoechst (blue) labels cell nuclei. Dashed lines indicate epidermal/dermal boundary of hair follicles. Scale bars represent 10 μm. Abbreviations: DP, dermal papilla; HF, hair follicle.

The absence of effects of laminin blockade on primary cilia formation and Shh responsiveness in human skin xenografts lead us to revisit the previously established relationship between LM511, primary cilia and Shh responsiveness in developing murine hair follicles. In particular, we examined whether lamα5-/- mice have discernible differences in known early dermal papilla markers compared to wild type mice. We first confirmed that lamα5-/- mice lack LM511 incorporation in the basement membrane zone. The antigen is apparent around hair follicles of control and absent from those in null mice (Figure 3A). We next compared the expression of several markers of developing dermal papilla in E16.5 mice [29-33]. For the early dermal papilla markers CD133, p75 and syndecan-1, we find similar expression in dermal condensates associated with Stage 1 and Stage 2 hair follicles in control and lamα5-/- animals (Figure 3C-E). In addition, we also quantified the number of p75-positive dermal cells per hair follicle in these mice and obtain similar results for Stage 1 and Stage 2 follicles in control and null animals. Our analysis, then, fails to show any abnormalities in the dermal papilla of lamα5-/- mice at E16.5.

**Figure 4:**
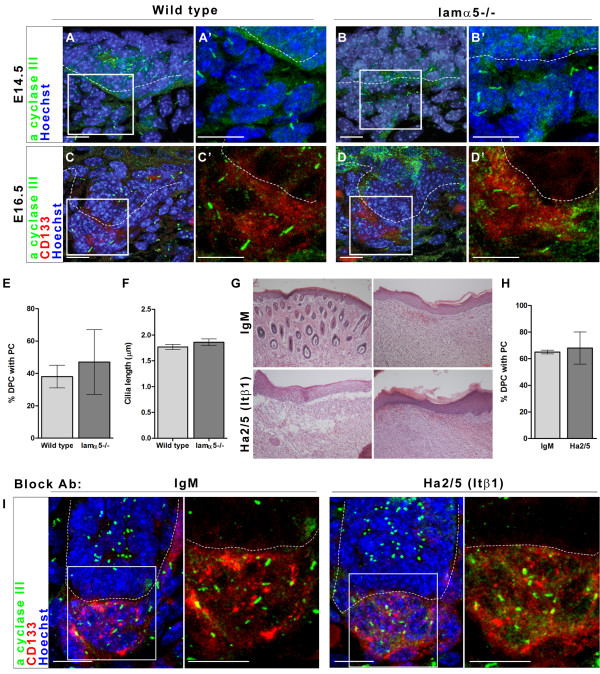
**Hair follicles in laminin alpha 5 null mice have primary cilia**. A-D: Confocal z-stacks of primary cilia and dermal papilla in hair follicles of E14.5 (A-B) and E16.5 (C-D) skin of wild type (A, C) and lamα5-/- (B, D) animals. Adenylyl cyclase III (green) indicates the primary cilia, CD133 (red) marks the dermal papilla cells and Hoechst dye (blue) indicates cell nuclei. A'-D': Zoom of boxed areas in A-D. E: Confocal z-stacks of primary cilia in dermal papilla of wild type E16.5 skin treated overnight with either control IgM or Ha2/5 antibody to Itβ1 (as indicated). Immunofluoresence same as in A-D. F: H&E stained frozen sections of 9-day allografts of wild type E16.5 skin treated overnight with either control IgM or Ha2/5 antibody (as indicated). IgM treated allografts show significant hair growth and normal hair-free skin. Ha2/5 treated allografts show both blistered and intact epidermis with little to no hair growth, indicating the ability of this antibody to block Itβ1 function and hair follicle development. G: Percent dermal papilla cells with primary cilia in z-stacks of dermal papilla from E16.5 skin treated with either IgM or Ha2/5 antibody (as in E). All scale bars indicate 10 μm. Abbreviations: DPC, dermal papilla cells; PC, primary cilia; Ab, antibody.

Similarly, we examined the presence of primary cilia in dermal papilla of E16.5 lamα5-/- mutants. We find that mutant mice do contain primary cilia in dermal condensates of developing hair follicles (Figure 4A-D). At E14.5, we observe primary cilia in dermal cells adjacent to presumptive placodes, identified as areas of epidermal thickening (Figure 4A-B). At E16.5, we identify the dermal papilla in Stage 2 follicles with CD133 and observe primary cilia in these cells (Figure 4C-D). The number of dermal papilla cells containing primary cilia was quantified in wild type and lamα5-/- mice at E16.5 and no significant difference was detected (Figure 4E). In addition, both wild type and null animals display cilia at 1.5-2.0 μm in length (Figure 4F). We conclude that in the absence of LM511 protein, dermal papilla differentiation and primary cilia formation proceed normally up to E16.5.

### Epithelial, but not dermal, Itβ1 is required for hair follicle epithelial invasion

A drawback of studies with lamα5-/- mutants is that LM511 is a secreted molecule that can associate with both epithelial and dermal cells, preventing definite conclusions as to the cell type in which it is required. Consequently we investigated the cell-autonomous requirement for the LM511 receptor, Itβ1, in dermal cells using both blocking antibodies and mouse mutants. Initially, we confirmed the effectiveness of the Itβ1 blocking antibody, Ha2/5 [34]. We soaked E16.5 wild type skin with control IgM and Ha2/5 antibody overnight and then grafted the treated skin onto the back of immuno-compromised (SCID) mice for continued growth. Grafted skin treated with control antibody formed stable stratified epithelia and regions of dense hair (Figure 4G, top panels). On the other hand, grafted skin first treated with Ha2/5 antibody to Itβ1 formed only a minimal patch of graft-derived hair (consisting of fewer than 10 hair follicles, data not shown) and some attached stratified epithelia. In large part, these treated grafts resulted in blistered graft skin, indicating that blocking Itβ1 function in the epithelial cells reduced the ability of the epidermis to remain associated with the basement membrane and form hair, which is in line with previous reports of epithelial Itβ1 function in skin (Figure 4G, lower panels) [22,23].

With this antibody capable of blocking Itβ1 function in murine skin, we treated wild type E16.5 whole backskin with Itβ1 blocking antibodies overnight, an experiment previously reported to eliminate primary cilia in dermal papilla cells [21]. Here, treated skin still contains primary cilia in both epidermal and dermal components of developing hair follicles (Figure 4I). In these tissues, both control treated and Ha2/5 antibody treated skin displayed approximately 65% of dermal papilla cells with primary cilia (Figure 4H). This result was consistent when a range of concentrations of blocking antibody was used, from 40 to 300 μg/ml (data not shown). This indicates that the Ha2/5 antibody is effective in blocking Itβ1 function, but does not reduce primary cilia in wild type skin. This corroborates our observation of the presence of primary cilia in the dermal papilla of lamα5-/- mice and argues against a role for LM511/Itβ1 in ciliogenesis.

To confirm our results with blocking antibodies, we examined skin function in a mouse lacking Itβ1 in the mesenchyme cells of the ventral and lateral skin. Mice expressing Prx-1 driven Cre-recombinase have been previously used to demonstrate a requirement for primary cilia components in hair follicle [15,35]. Thus, we reason that if LM511 association with Itβ1 is required in dermal cells for primary cilia formation/maintenance or proper Shh signaling, conditional deletion of Itβ1 in this cell population should also negatively affect hair follicle morphogenesis.

**Figure 5:**
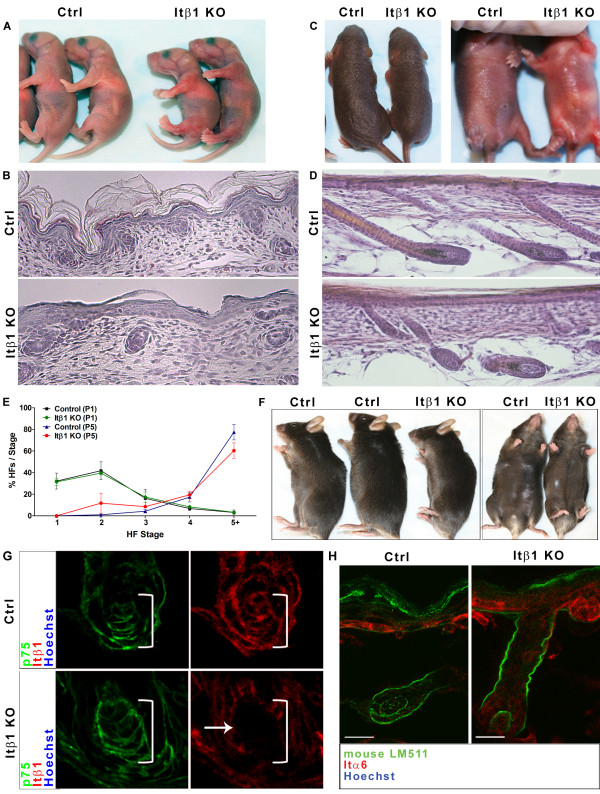
**Prx1-Cre; Itβ1^flox/flox ^mice exhibit normal hair follicle morphogenesis and post-natal cycling**. A: Mixed littermates (Ctrl) and Prx1-cre; Itβ1^flox/flox ^(Itβ1 KO) mice at 1 day post-natal (P1). Conditional knockouts display slightly reduced size and a reddened appearance on ventral and lateral surfaces. B: Representative H&E stained skin sections from the ventral skin over the sternum of P1 control and conditional knockout mice. C: Control and conditional knockout mice at 5 days post-natal (P5) showing the reduced size and reddened ventral skin of mutant mice. D: H&E stained skin sections from the ventral skin of P5 control and conditional knockout mice showing hair follicles in advanced stages. E: Quantitation of hair follicle stages in P1 and P5 control and conditional knockout mice showing similar patterns of hair development. F: Control and conditional knockout mice at 82 days showing the normal appearance of coat on the ventral and lateral regions. G: Immunofluoresence for p75 (green) and Itβ1 (red) in sections of ventral skin from P1 control and conditional knockout mice at P1 (as indicated). Brackets indicate p75+ dermal papilla. Note lack of Itβ1 signal in the dermal papilla of a Stage 4 hair follicle (arrow). H: Immunofluoresence for mouse LM511 (green) shows normal incorporation of this laminin in P1 control and conditional knockout mice. Scale bars represent 20 μm. Abbreviations: Ctrl, control; Itβ1 KO, dermal Itβ1 knockout; HFs, hair follilces.

Indirect immunofluorescence for Itβ1 in ventral/lateral skin of P1 Prx1-Cre; Itβ1^flox/flox ^mice indicates that these late stage hair follicles do not express Itβ1 in dermal papilla cells (for a reference to Itβ1 antibody staining in mouse skin see [22]). In control mice, immunofluorescence for Itβ1 shows cell surface expression of Itβ1 on basal keratinocytes, hair follicle epithelial cells and dermal papilla cells (Figure 5G, top panels and Additional File 2, Figure S2). This expression pattern partially overlaps with that of p75 which is found on the cell surface of dermal papilla, suprabasal keratinocytes and in hair follicle epithelial cells in these late-stage follicles. On the other hand, ventral hair follicles in conditional knockouts contain p75-positive dermal papilla cells void of Itβ1 expression (Figure 5G, bottom panels). In skin of these mice, Itβ1 expression still overlaps with p75 expression in hair follicle epithelial cells (Additional File 2, Figure S2). We also demonstrate that conditional deletion of dermal Itβ1 does not disrupt localization of LM511. As in control skin, LM511 is observed in dermal Itβ1 knockouts incorporated into the basement membrane zone of interfollicular epidermis and hair follicles and is especially pronounced in the basement membrane zone surrounding follicles (Figure 5H). This indicates that conditional deletion of dermal Itβ1 does not disrupt the localization of LM511 in developing hair follicles and that the formation of the basement membrane zone is not driven by dermal Itβ1 but by epidermal Itβ1 only [22,23]. We additionally confirmed that deleting Itβ1 from dermal cells does not disrupt additional components of the basement membrane zone or extracellular matrix around endothelial structures. Immunolocalization of tenascin C, fibronectin, perlecan, collagen I and collagen IV are all comparable in P1 skin of control and conditional knockouts (Additional File 3, Figure S3A-E).

At post-natal day 1 (P1), Prx1-Cre; Itβ1^flox/flox ^mice look relatively normal except for reddened skin in the areas of expected Prx1-Cre expression (Figure 5A, [35]). We currently do not know the cause of this reddened appearance, as histology does not indicate inflammation in the skin of these mice (Figure 5B). Histological examination of ventral skin above the sternum in conditional knockouts reveals hair follicles in stages varying from placode to Stage 2 peg (Figure 5B). Analogously, P5 mice maintain the reddened appearance and small size of P1 mice. Like control mice, the ventral skin of conditional knockout mice at P5 contains abundant fully developed hair follicles. Quantification of the stages of hair follicles in P1 and P5, control and knockout mice indicates comparable developmental patterns (Figure 5E). Post-natal hair cycling is also normal in knockout mice, as adult mutants have normal hair coats in knockout regions (Figure 5F).

**Figure 6:**
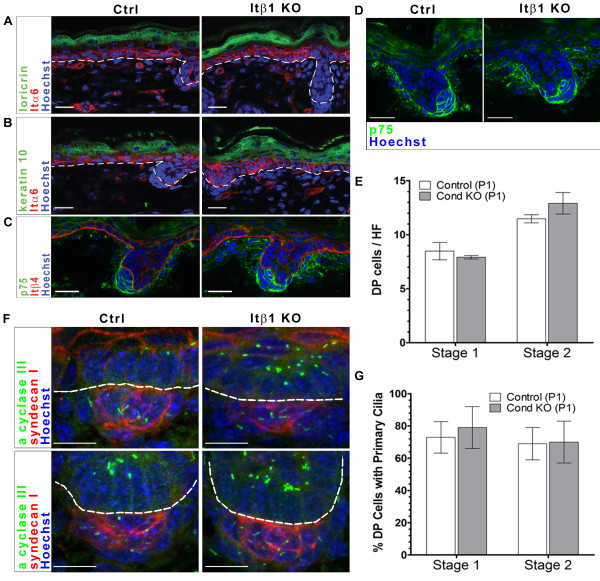
**Dermal condensates in Prx1-Cre; Itβ1^flox/flox ^mice appear normal and contain primary cilia**. A: Immunofluoresence for loricrin (green) in P1 control and conditional knockout mice, showing similar staining of the statrum granulosum. B: Immunofluoresence for keratin 10 (green) in P1 control and conditional knockout mce, showing similar staining of the straturm spinosum Itα6 staining in A-B shows normal basal cell layers. C: Immunofluoresence for Itβ4 (red) and p75 (green), showing normal levels of Itβ4 staining in the epithlelia. D: Immunofluoresence for p75 (green) in P1 control and Prx1-Cre; Itβ1^flox/flox ^mice showing similar dermal papilla expression patterns. A-D: Scale bars indicate 20 μm E: Number of dermal papilla cells in condensates of Stage 1 and 2 hair follicles of P1 control and Prx1-Cre; Itβ1^flox/flox ^mice. F: Confocal z-stacks of immunofluoresence for primary cilia and dermal papilla in Stage 1 (top panels) and Stage 2 (bottom panels) hair follicles of P1 control and Prx1-Cre; Itβ1^flox/flox ^mice. Adenylyl cyclase III (green) marks the primary cilium shaft, syndecan I (red) indicates dermal papilla cells and Hoechst dye (blue) indicates cell nuclei. Scale bars indicate 10 μm G: Percent dermal papilla cells with a primary cilium in Stage 1 and 2 hair follicles of P1 control and conditional knockout mice. All dotted lines indicate the epidermal/dermal border. Abbreviations: Ctrl, control; Itβ1 KO, dermal Itβ1 knockout; DP, dermal papilla; HF, hair follicle.

In order to confirm that deletion of dermal Itβ1 does not have discernable effects on skin development, we determined the expression of epithelial differentiation markers loricrin and keratin-10 in skin of conditional knockouts. Loricrin expression is found in the stratum granulosum of properly differentiated epidermis. This expression pattern is evident in control and Prx1-Cre; Itβ1^flox/flox ^skin (Figure 6A). Similarly, conditional knock-outs display a normal expression pattern of keratin-10, which localizes to the stratum spinosum and granulosum of the epidermis (Figure 6B). Deletion of epithelial Itβ1 causes reduced presence of integrin alpha-6 and integrin beta-4 in associated basement membrane zone [22]. This is not the case in Prx1-Cre; Itβ1^flox/flox ^mice as the expression of these integrins is not altered in basolateral membranes of basal keratinocytes (Figure 6B-C). As in lamα5-/- mice, dermal papilla markers appear normal in hair follicles of Prx1-Cre; Itβ1^flox/flox ^mice. We tested expression of p75 and syndecan-1 in conditional knockouts and find expression domains and levels similar to those in control mice (Figure 6D and data not shown). We also determine that the number of p75-positive dermal papilla cells per hair follicle in dermal Itβ1 knockouts is comparable to that in control mice at P1 in both Stage 1 and Stage 2 hair follicles (Figure 6E). In addition, we confirmed the presence of primary cilia in dermal papilla of hair follicles in Prx1-Cre; Itβ1^flox/flox ^skin (Figure 6F). We quantified the presence of primary cilia in Stage 1 and Stage 2 hair follicles at this time point and find no difference in the presence of cilia in control versus conditional knockout animals (Figure 6G). This indicates that dermal Itβ1 is not required for the formation of primary cilia in dermal cells or the condensation of dermal cells into dermal papilla. Therefore, unlike deletion of Itβ1 in epithelial cells, which has been shown to be necessary for proper skin and hair formation, deletion of Itβ1 from dermal cells does not impact this tissue.

## Discussion

In developing skin, laminins have been reported to act via their integrin receptors on both the epithelium as well as the underlying dermal organizer. Using a human model of skin epithelium, we have shown that inhibition of laminin-511 and 332 function prevents epithelial invagination, but leaves the primary cilia and Shh target gene induction apparently intact. Consistent with a primary effect on the epithelium, few abnormalities derive from removing the laminin receptor Itβ1 in dermal cells, thus clarifying how laminin-511 functions in the skin.

### LM511 and LM332 in primary cilium and Shh signaling

The primary cilium has quickly garnered attention as a sensory organelle required for correct processing of Shh signal reception during hair follicle development and tumorigenesis. A previous model suggested that the structure of the primary cilia in the dermal papilla dependended on extracellular cues from the basement membrane zone through LM511 binding to dermal Itβ1 [21]. Our current work, however, argues against a direct role for laminins 511 or 332 in ciliogenesis or Shh target gene induction. These differences may be, in part, due to a previous lack of precise aging of embryonic mice, which may have resulted in the inadvertent study of degenerating hair follicles and skin after the mutant animals began to be reabsorbed in utero. In addition, the use of mouse anti-human acetylated tubulin antibody to detect cilia, which is sub-optimal in mouse skin due to extensive background staining of anti-mouse secondary antibodies, may have exacerbated these problems. These are difficulties we believe we have overcome through careful mouse breeding and the optimization of primary cilia staining protocols. We believe our conclusions regarding the lack of a role for LM511 and Itβ1 in the dermal papilla are accurate because of the technique optimization, and the corroboration of our results from analysis of the dermal Itβ1 mutants. Recent studies from our lab and others have shown the important role of actin and the microtubule cytoskeleton in maintaining cilium structure [16,36,37]. However, our current results reiterate the paucity of information known about ciliogenesis and the extracellular cues that regulate cilium function.

In our human BCC xenograft model, blocking both LM511 and LM332 is required to inhibit tumor invasion, while blocking of LM511 alone has no discernable effect. These results sharply contrast with the inhibition of LM511 in human hair follicle xenografts, which dramatically inhibits hair follicle epithelial invasion using comparable doses of blocking antibody in a similar experimental setting [20]. We have thus uncovered a potentially important difference between hair follicle and BCC epithelial growth. In invaginating hair follicle epithelium, LM511 is the predominant laminin supporting hair follicle growth, while in BCCs LM332 appears to be sufficient to compensate for loss of LM511. Interestingly, recent functional studies reinforce the differences between hair follicle and BCC epithelium. Forced activation of the Shh pathway in interfollicular epithelium, but not hair follicle epithelium induces BCC, suggesting that coexpression of LM511 and LM332 seen in the interfollicular epithelium may offer a selective advantage for invading tumors [38].

### LM511 and Itβ1 in hair follicle morphogenesis

The presence of normal late stage hair follicles in Prx1-Cre; Itβ1^flox/flox ^mice indicates that dermal Itβ1 signaling is not required for proper hair follicle morphogenesis. Conditional knockout of epithelial Itβ1, on the other hand, clearly constitutes a requirement for epithelial Itβ1 in skin and hair development [22,23]. This may be partially due to the role of Itβ1 in the formation of a stable basement membrane zone. LM511 is also important for basement membrane zone stability, as lamα5-/- null mice contain a discontinuous lamina densa specifically in association with hair follicles [20,22,23]. The importance of the basement membrane zone for epithelial downgrowth is also indicated by the lack of invagination after LM511and LM332 inhibition in our human xenograft model of BCC.

Previous studies of lamα5-/- mice also noted the reduced expression of *SHH *and *GLI1 *transcripts by RNA in-situ in epithelia of E16.5 hair follicles [20]. This indicates that LM511 may have an indirect effect on the expression of Shh ligand, and that this effect may represent one aspect of the more extensive skin phenotype in epithelial Itβ1 mutants. Given the requirement of reciprocal signaling during morphogenesis, we speculate that reduced Shh signaling in the hair follicles of lamα5-/- mice may be a result of failed association of epithelial cells with the basement membrane zone and/or reduced expression of Shh ligand in hair follicle epithelia, which eventually results in degeneration of both follicle and dermal papilla. Therefore, our results and previously published data lead us to assert a primary effect of LM511 function on epithelial cells. We cannot yet exclude the possibility that LM511 may function via an unidentified dermal papilla receptor, but current available data does not support this alternative.

## Conclusions

• LM511 and LM332 are required for tumor invagination in a xenograft model of human BCC, but are not required for Shh target gene induction or primary cilia formation.

• Lamα5-/- mice exhibit a stage-specific defect in hair follicle morphogenesis. Stalled hair follicles contain dermal papilla with normal cell numbers, early marker expression and cilia.

• Deletion of dermal Itβ1 does not affect hair follicle morphogenesis or post-natal hair cycling. Itβ1-null follicles contain dermal papilla with normal cell numbers, early marker expression and primary cilia

• These results suggest that LM511 may participate in hair follicle morphogenesis by acting on the hair follicle epithelium, perhaps in part by facilitating epithelial cell invagination or regulating Shh ligand expression.

## Methods

### Antibodies

Hybridomas for mouse mAb 4c7 and BM165 to LM511 and the laminin alpha 3 chain, respectively, were provided by Dr. M. P. Marinkovich and isolated according to previous reports [27,39]. Rabbit pAb to LM332 (pKaL), mouse mAb to Itβ1 (p5D2) and rabbit anti-sera to mouse laminin α5 were generous gifts of Dr. M. P. Marinkovich (Stanford, CA). Additional antibodies used were as follows: 3E1 (MAB1964), collagen VII (MAB2500), integrin alpha-6 (MAB1378) and integrin beta-1 (MAB1997) from Millipore (Billerica, MA); involucrin (ab53112), fibronectin (ab23750), perlecan (ab44937), collagen I (ab34710), collagen IV (ab19808) and tenascin C (ab6346) from Abcam (Cambridge, MA); acetylated alpha tubulin (T6793) and control mouse IgG (I8765) from Sigma (St. Louis, MO); adenylyl cyclase III (sc-588) and rootletin (sc-67824) from Santa Cruz Biotechnology (Santa Cruz, CA); human cytokeratin 10 from Dako (Denmark); keratin 14 (PRB-155P), mouse keratin 10 (PRB-159P) and loricrin (PRB-145P) from Covance (Princeton, NJ) and Ki67 (clone SP6) from Neomarker (Fremont, CA).

### Human skin xenografts

Xenografts were conducted as previously described [25]. For experiments receiving a single antibody (IgG, 4c7 or BM165) animals received intraperitoneal injections carrying 1.0 mg of antibody every week starting in the first week of growth. In a preliminary experiment, we determined that blocking grafts with both LM511 and LM332 antibodies in the first week of growth resulted in severe blistering, so we began injections in the second week of growth. All studies involving mice were performed in accordance with the policies of the Stanford IUPAC.

### Immunohistochemistry

Detection of primary cilia in 12-μm sections of paraffin embedded tissues involved antigen retrieval with 1 mM EDTA (pH 8.0). Blocking buffer contained 20% NHS with 0.1% Triton X-100 and staining solution contained 5% NHS/0.1% Triton X-100. The rest of the protocol was standard. For all other immunofluoresence (immunofluoresence) on paraffin embedded tissues, 5-10 μm sections were treated as above except that antigen retrieval was performed with 10 mM Citrate. For immunofluoresence on frozen tissues, 8 μm sections were fixed with 4% PFA and subjected to standard staining protocols with a blocking/staining solution consisting of 5% NHS, 0.1% Triton X-100 in PBS. Alexa Fluor (Invitrogen) secondary antibodies were used at 1:1000 and Hoechst dye was used at 1:10,000. ProLong Gold Antifade reagent (Invitrogen) was used for mounting. TUNEL assays were conducted according to the TUNEL TMR-Red Assay kit (Roche, Switzerland). Fluorescent imaging was performed on either a Zeiss Axioskop with Openlab software or with the Zeiss LSM 510 with Ti:Sapphire laser for 2-photon excitation (maintained by the Cell Sciences Imaging Facility at Stanford University). Confocal images and z-stacks were processed with Zeiss LSM Image Browser and/or Volocity (Improvision) software packages and Photoshop CS2.

### DIG RNA in-situ hybridization

RNA in-situ hybridization was performed on frozen preparations of xenograft tissues as previously described [40].

### Mice

All mouse studies were performed in accordance with the policies of the Stanford IUPAC. For experiments involving WT embryonic mice, C57Bl6 mice were mated and 12 noon on the day plugs were observed was considered embryonic day 0.5 (E0.5). Lamα5 knock-out mice were maintained and genotyped as described previously [41]. Prx1-Cre mice were purchased from Jackson Labs. Itβ1^flox/flox ^mice were obtained from Dr. Scott Kuwada with the University of Utah and have been previously described [22]. For the conditional knockout of Itβ1 in lateral mesenchyme, Prx1-Cre; Itβ1^flox/+ ^males were mated to Itβ1^flox/flox ^or Itβ1^flox/+ ^females. The presence of the Prx1-Cre transgene was determined with primary ciliaR for Cre.

## Authors' contributions

MCD designed experiments; carried out immunofluorescence, RNA in-situ, histological mouse phenotype and statistical analyses; participated in study coordination and drafted the manuscript. HZ helped to design and then carried out all skin grafts. ST helped to design and then carried out primary cilia immunofluorescence. SW helped to design and then carried out immunofluorescence on graft tissues and carried out mouse husbandry and breeding. MPM helped to conceive the study. AEO conceived of the study, participated in its design and coordination and helped to draft the manuscript. All authors read and approved the final manuscript

## Supplementary Material

Additional file 1 **Figure S1: Blockade of LM511 and LM332 in *SHH*-expressing grafts does not affect the proliferation of cells expressing SHH ligand**. Percent Ki67-positive cells of total basal or suprabasal cells in human skin xenografts carrying either control *GFP *or *SHH *retrovirus and treated with control IgG, 4c7 antibody against LM511 or BM165 antibody against LM332, as indicated. Quantitation corresponds to representative images presented in Figure 1E. Click here for file

Additional file 2 **Figure S2: Prx1-Cre; Itβ1^flox/flox ^mice have specific deletion of Itβ1 in dermal cells but not epithelial or endothelial cells**. Immunofluoresence for p75 (green) and Itβ1 (red) in sections of ventral skin from P1 control and conditional knockout mice at P1 (as indicated). Zoom images in Figure 5G are extracted from these images. Click here for file

Additional file 3 **Figure S3: Deletion of dermal Itβ1 does not disrupt skin differentiation, BMZ components or endothelial structures**. Immunofluoresence for various markers in skin of control and conditional knockout mice. A: Loricrin (green) and Itα6 (red) indicate granule and corneal cell layers and basal cell layers, respectively, of interfollicular epidermis. B: Keratin 10 (green) indicates the prickle and granule cell layers of interfollicular epidermis. C-D: IF for tenascin C (red) indicates a normal lack of this marker in early dermal papilla and presence in the rest of the dermis. D: IF for fibronectin (green) indicates a normal lack of this marker in dermal papilla and presence in ECM in non-DP dermis. E: IF for perlecan (red) indicates normal endothelial structures. F-G: No differences are seen in collagen I (F, green) and collagen IV (G, green) expression in skin of control and conditional knockouts. Click here for file
