# Supplementary figures and images for: Laminin-511 and integrin beta-1 in hair follicle development and basal cell carcinoma formation
Source: BMC Dev Biol. 2010 Nov 10;10:112. doi: 10.1186/1471-213X-10-112 (PMC2995472; doi:10.1186/1471-213X-10-112)

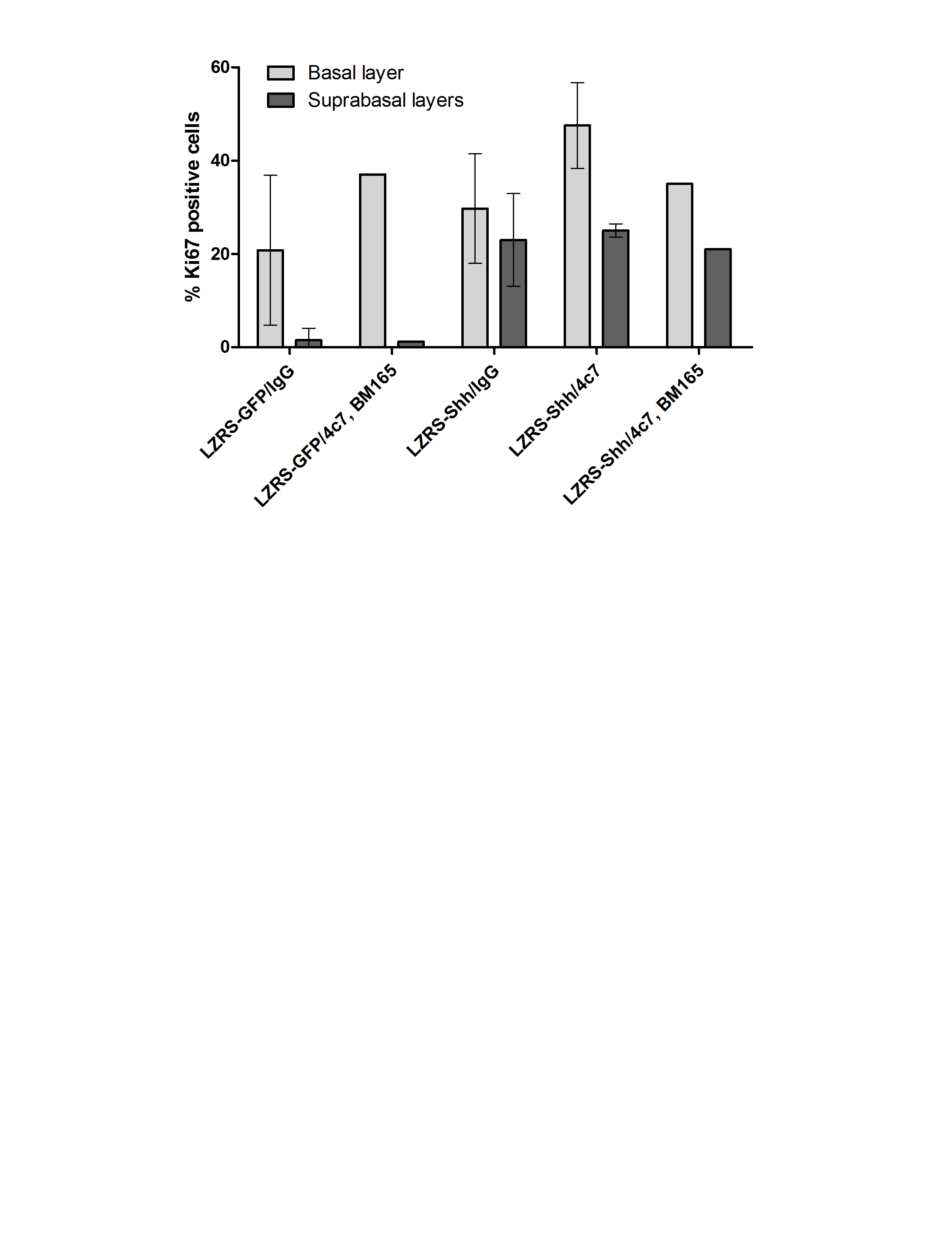

Supplement: Additional file 1 — Figure S1: Blockade of LM511 and LM332 in SHH-expressing grafts does not affect the proliferation of cells expressing SHH ligand. Percent Ki67-positive cells of total basal or suprabasal cells in human skin xenografts carrying either control GFP or SHH retrovirus and treated with control IgG, 4c7 antibody against LM511 or BM165 antibody against LM332, as indicated. Quantitation corresponds to representative images presented in Figure 1E. [file 1471-213X-10-112-S1.JPEG]

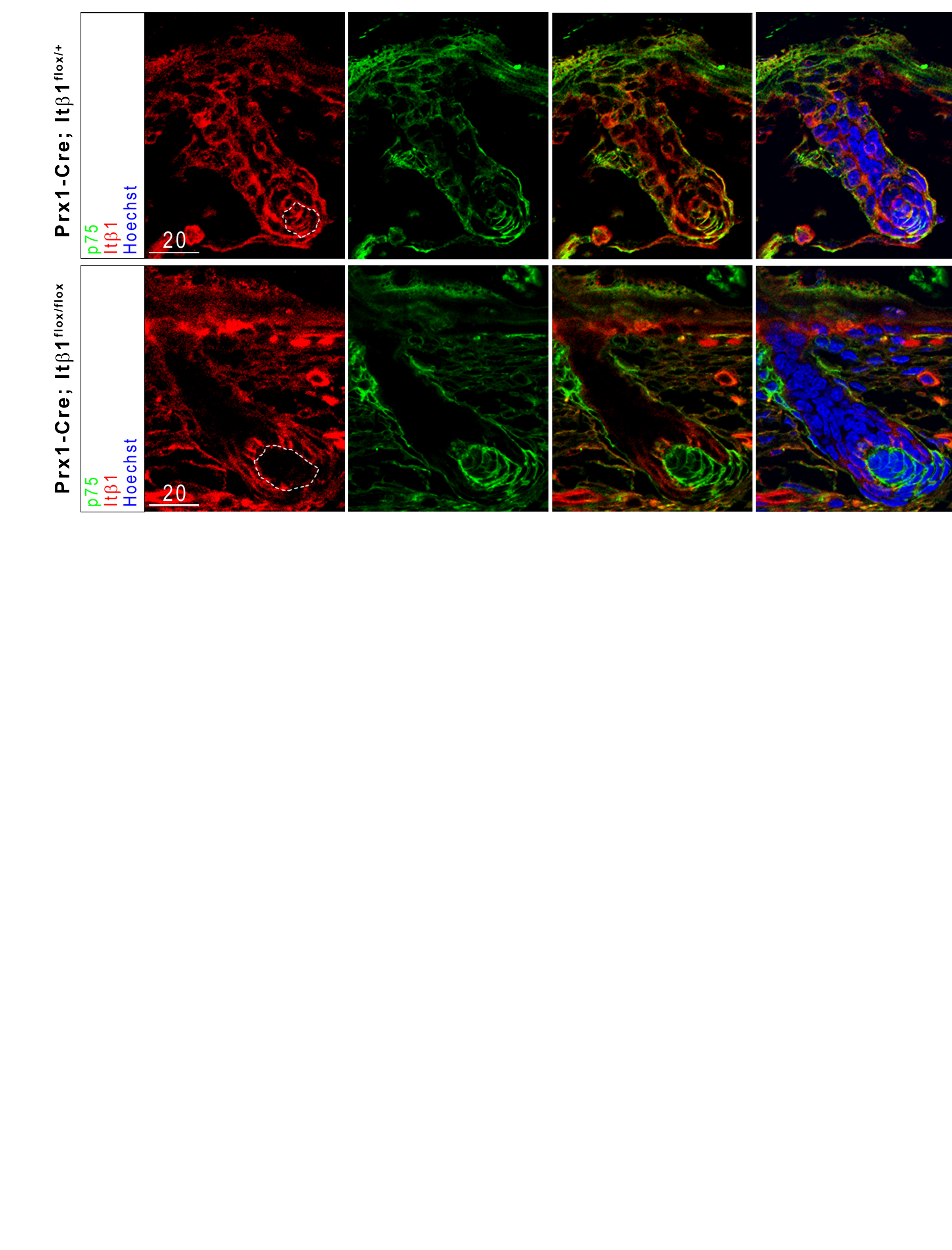

Supplement: Additional file 2 — Figure S2: Prx1-Cre; Itβ1flox/flox mice have specific deletion of Itβ1 in dermal cells but not epithelial or endothelial cells. Immunofluoresence for p75 (green) and Itβ1 (red) in sections of ventral skin from P1 control and conditional knockout mice at P1 (as indicated). Zoom images in Figure 5G are extracted from these images. [file 1471-213X-10-112-S2.JPEG]

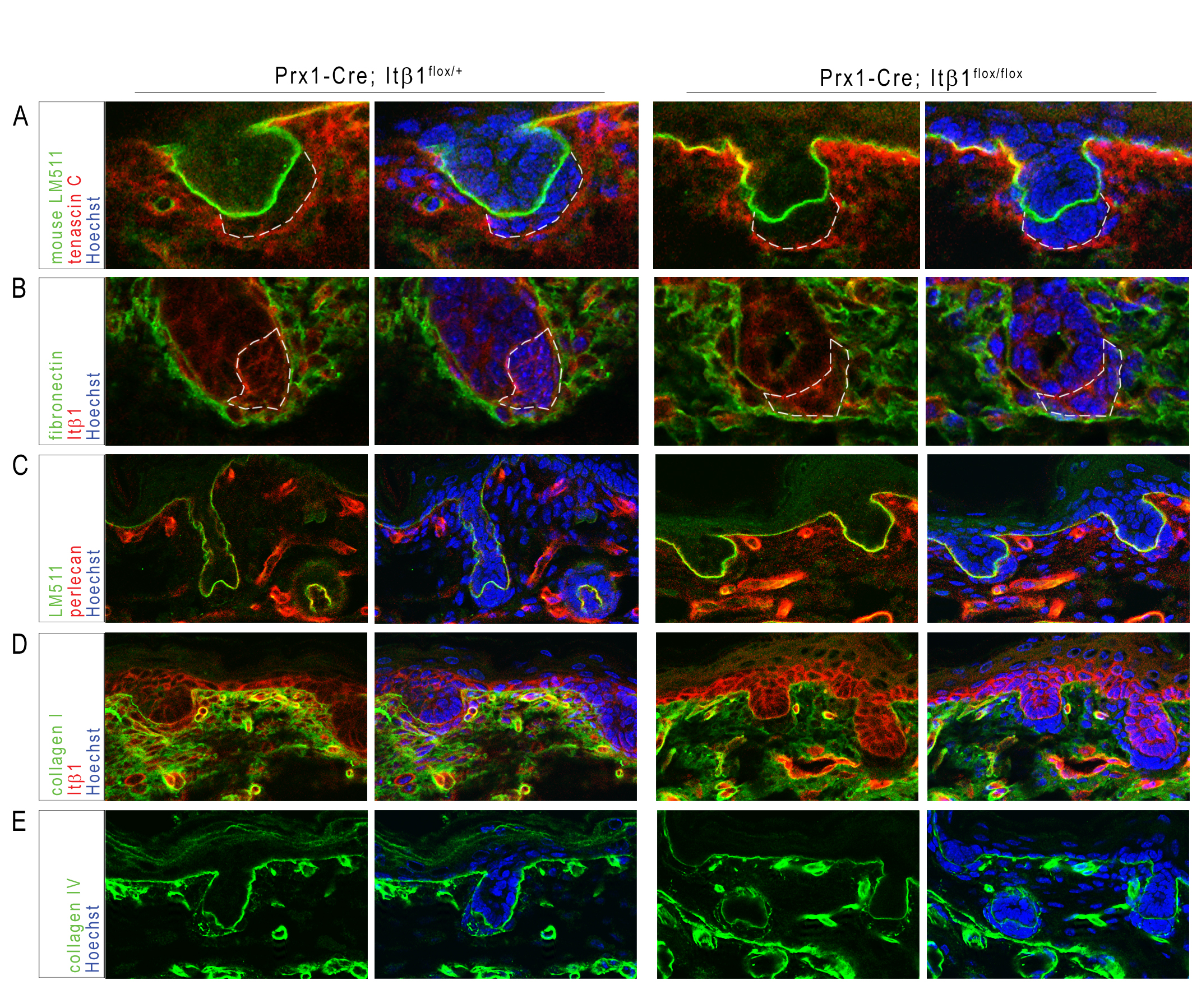

Supplement: Additional file 3 — Figure S3: Deletion of dermal Itβ1 does not disrupt skin differentiation, BMZ components or endothelial structures. Immunofluoresence for various markers in skin of control and conditional knockout mice. A: Loricrin (green) and Itα6 (red) indicate granule and corneal cell layers and basal cell layers, respectively, of interfollicular epidermis. B: Keratin 10 (green) indicates the prickle and granule cell layers of interfollicular epidermis. C-D: IF for tenascin C (red) indicates a normal lack of this marker in early dermal papilla and presence in the rest of the dermis. D: IF for fibronectin (green) indicates a normal lack of this marker in dermal papilla and presence in ECM in non-DP dermis. E: IF for perlecan (red) indicates normal endothelial structures. F-G: No differences are seen in collagen I (F, green) and collagen IV (G, green) expression in skin of control and conditional knockouts. [file 1471-213X-10-112-S3.JPEG]
